# The sound symbolism of food: the frequency of initial /PA-/ in words for (staple) food

**DOI:** 10.1515/ling-2021-0127

**Published:** 2023-01-27

**Authors:** Ian Joo

**Affiliations:** Department of Chinese and Bilingual Studies, The Hong Kong Polytechnic University, Kowloon, Hong Kong

**Keywords:** food, iconicity, sound symbolism

## Abstract

In different languages around the world, morphemes representing the (cooked form of) staple food or food in general tend to begin with a [+labial] phoneme followed by a [+low] phoneme (/pa-/, /ma-/, /fa-/, /wa-/, etc.). This article provides evidence for this phonological similarity by analyzing 66 sample languages’ morphemes representing the staple food within the society where each language is spoken. About a fourth of the morphemes referring to staple food begin with a [+labial] first phoneme followed by a [+low] second phoneme, which is a much higher proportion compared to another list of basic morphemes in the same 66 languages. I further argue that the motivation for this crosslinguistic tendency is the iconic association between the mouth-opening gesture and the concept of eating.

## 1 Introduction

Although there is no consensus on how to strictly define ‘staple food’ (Santich 1990), the term is generally employed to refer to the type of food that a community consumes on a daily basis and considers to be a principal dietary element in their culture. There is often a single dominant type of staple food that sustains a diet of a people: rice, for instance, is eaten daily in many East Asian cultures. In some cultures, several staple foods co-exist: most European countries today no longer rely on bread as the one and only staple food, but rather consume a variety of staple foods, such as pasta or potatoes.

The staple food can be a major part of a people’s diet, generally forming 30–70% of a population’s energy intake (Wheeler 1990). Moreover, it often plays a symbolic role within a culture. The well-known Russian custom of ‘bread and salt’ (*khl’eb-sol’* хлеб-соль), where guests are greeted with a loaf of bread and salt as a sign of hospitality, reflects the cultural importance Russians place on bread, their traditional staple food.

Since a language carries the culture of its speakers, a staple food often demonstrates its cultural significance in the language of the people it feeds. In a society where a single type of staple food is dominant, the term for that staple food is often synonymous with the term for food in general. Thus, the Korean word for cooked grain (*pap* 밥), the Korean staple food, can also refer to a meal: a Korean speaker can say that they have eaten *pap* even after having eaten pizza for lunch.

In this study, I shed light on a strikingly consistent phonological pattern in how different languages express the staple food of its speakers or food in general: the pattern of the initial phoneme being [+labial] and the second phoneme [+low], e.g., /pa-/, /fa-/, /ma-/, /wa-/, and so on, abbreviated as /PA-/. Several studies have shown that the phonological forms of lexemes representing certain meanings tend to include certain sounds: for example, morphemes that stand for a round object (such as ‘knee’) tend to bear phonemes with the [+round] feature (Blasi et al. 2016; Johansson et al. 2020; Joo 2020), the round shape being iconically represented by the lip-rounding articulation of the [+round] phonemes. In a similar vein, I propose that morphemes representing the staple food of the speakers of the respective language tend begin with /PA-/, motivated by the iconic resemblance of the articulation of /PA-/ and the gesture of opening the mouth to express eating.

## 2 The research question

I wish to verify the hypothesis that in a given set of sample languages, the morpheme representing the staple food of each language’s culture (or food in general, in the case where there is no single prominent staple food) tends to begin with /PA-/ significantly more often than average. Many studies have demonstrated crosslinguistic iconic patterns in the basic vocabulary of spoken languages, such as words for body parts or personal pronouns (Blasi et al. 2016; Gordon 1995; Johansson et al. 2020; Johansson and Zlatev 2013; Joo 2020; Nichols and Peterson 1996; Tanz 1971; Urban 2011; Woodworth 1991). The goal of this article is to test whether the morphemes representing the different staple foods of the world also show a crosslinguistic iconic pattern.

## 3 Methodology

What do I mean by ‘staple food’? What food consumed by whom in which period of time? In this research, I collected the morphemes standing for the nutritional source that is eaten daily or near-daily, supplying a significant portion of energy intake, and regarded as ‘the staple food’ (or ‘the principal food’, etc.) either by the members of the culture themselves or a description of their culture. The morpheme must refer to the default cooked form (e.g., ‘bread’) and not the raw source (e.g., ‘wheat’). Only in the cases where there is no distinct morpheme for the cooked form or no default cooked form have I chosen the morpheme that refers to the raw source. The morpheme must refer to what has been the single dominant staple food in the most recent tradition, as made evident by cultural or linguistic traits. For example, bread is no longer the single dominant staple food in contemporary Spain, as it competes with rice and pasta. However, Spanish proverbs that survive to this day, such as *nacer con un pan debajo del brazo* ‘to be born with a loaf of bread under the arm’, indicate the traditional status of bread as the most important staple. By specifying ‘the most recent tradition’ I exclude other foods that may have been the staple food in earlier traditions (e.g., the Japanese staple food before the advent of rice agriculture in Japan). When such historical information is not available, I have chosen what is simply referred to as ‘the staple food’ or the like.

The 66 sample languages are those used in Joo’s (2020) typological study on lexical iconicity. In Joo’s study, the largest language (in terms of native speaker population) of each of the largest 66 language families (in terms of speaker population) was selected as a sample language. For example, Spanish (1st sample) is the largest language of the Indo-European family, which is the largest language family, and Mandarin (2nd sample) is the largest language of the Sino-Tibetan family, which is the 2nd largest language family, and so on. From each of these 66 languages, Joo compiled the Leipzig-Jakarta List (Tadmor 2009), a list of 100 basic meanings.

In each of the 66 sample languages, I searched for the morpheme that refers to the staple food, or the default cooked form of it, if a language has a separate morpheme for the default cooked form (I chose the morpheme for the uncooked staple food if there is no single dominant cooked form). In cases where the morpheme for ‘food’ or ‘meal’ is identical to the (cooked form of) staple food in a language, I selected that morpheme. When a language is spoken in a society where there is no single dominant type of staple food, I chose the morpheme for ‘food’. When there is no single morpheme for ‘food’, I have chosen the morpheme for ‘to eat’.

Table 1 shows the sample language morphemes referring to staple food. The morphemes were transcribed in the International Phonetic Alphabet, excluding tone and stress.

**Table 1:** Staple food morphemes.

Language	Morpheme	Meaning
Spanish	**pan**	Bread
Mandarin Chinese	**fan**	Cooked grain
Yoruba	iʃo	Yam (Bascom 1951: 44)
Egyptian spoken Arabic	ʕeːʃ	Bread
Javanese	səɡɔ (Suharno 1982: 159)	Cooked rice
Telugu	bijjan	Rice
Turkish	ekmek	Bread
Japanese	haɴ	Cooked grain
Vietnamese	kʌm	Rice
Thai	kʰaːw	Rice
Korean	**bab**	Cooked grain
Dholuo	kuon	Porridge (Ocholla-Ayayo 1980: 131)
Hungarian	kɛɲeːr	Bread
Chuanqiandian Cluster Miao	tɕua (Wang 1985: 175)	Cooked grain (Zhongguo Kexueyuan Minzu Yanjiusuo Guizhou Shaoshu Minzu Shehui Lishi Diaocha Zu 1963: 264)
South Bolivian Quechua	**papa**	Potato (Krögel 2015: 24)
Peripheral Mongolian	**max**	Meat
Kabardian	**pˀastə**	Pudding (Jaimoukha and Malherbe 2009: 130)
Kʼicheʼ	**wa**	Food (corn or in general) (Christenson 1993)
Paraguayan Guarani	**mad**^n^**iʔo** (Dávalos de Céspedes et al. 2015)	Cassava (Grubb 2011: 77–78)
Georgian	p’uri	Bread (in eastern Georgia) (Watson 1994: 132)
Enga	awamu (Lang 1978)	Sweet potato (Clark 1978: 92–107; Waddell 1975: 252)
Eastern Huasteca Nahuatl	−^a^	Tortilla (John J. Sullivan, p. c.)
Central Aymara	tʃʼuɲu (Ajacopa 2020)	chuño (freeze-dried potato) (Reclus 1894: 389)
Mezquital Otomi	hme (Néstor Hernández Green, p. c.)	Tortilla (Néstor Hernández Green, p. c.)
Wayuu	uːxoɺu	Chicha (Juan Esteban Torres Muriel and Claudia Patricia Puerta Silva, p. c.)
Basque	oɡi	Bread
Ngäbere	?	Vegetable (Visser 2021: 8)
Highland Totonac	tʃux	Tortilla (Beck 2016: 11)
Khoekhoe	kup	Mutton (Percival 1969: 262)
Galela	ino (Ipol et al. 1989: 94)	Food (Ishige 1978: 239)
Mapudungun	ɪn	To eat (Dillehay 1998: 219)
Western Highland Purepecha	itʃuskuta (Chamoreau 2008: 474)^b^	Tortilla (Amerlinck 1995: 59)
Woods Cree	miːts-	Eat (Curtis 1928: 62–63)
Navajo	**pɑːx**	Bread (Deogaonkar and Deogaonkar 2002: 108)
Highland Popoluca	aːɲi	Tortilla (Wauchope and Vogt 2015)
Ambulas	mu	Thing, food (Clark 1978: 273; Scaglion 2017)
Mískito	jaura	Cassava (Dale Terry et al. 1979: 124)
Shuar	**mama**	Cassava (Pillsworth 2008)
Northern Emberá	**pʰata** (Mortensen 1999: 117)	Plantain (Kane 2004: 220; Mortensen 1999: 117)
Bukiyip	kakwitʃ	Food (Clark 1978: 297)
Northwestern !Kung	‼ʔʰaũ	Meat (Fernandes-Costa et al. 1984)
Greenlandic	tamɔ	Eat (Vahl et al. 1928: 202)
Burushaski	hari	Barley (Lorimer 1938: 10)
Sentani	fi	Sago (Yamamoto et al. 2020)
Terei	tamu	Food; To eat (Clark 1978: 3)
Macushi	jaʔɾe (Carson 1982: 135)	Meat (Carson 1982: 135)
Ap Ma	suboɡ (Pryor 1990: 22)	Sago core (Clark 1978: 268)
Páez	kaʔka	Potato (Evans-Pritchard 1973)
Wichí Lhamtés Vejoz	**fʷaʔa** (Nercesian 2017)	Carob (Mariani et al. 2017: 287; Occhipinti 2005: 136)
Sandawe	nua	Porridge (Newman 1975)
Xibe	buda	Food (He and Tong 1994)
Toba	?	?
Ticuna	tʃoʔni	Fish (Nimuendajú et al. 1971 [1952])
Kaingang	**faɡ**	Pine nut (Becker 1991)
Pitjantjatjara	**mai**	Non-meat food (Also ‘food’ in general, e.g., Love 1945: 76)
Guahibo	neβajɯ	Bitter cassava (Rojas 1994)
Shipibo-Conibo	toβã	Cooked cassava (Lathrap 1976: 198)
Yanomamö	kuratʰa	Cooking banana (Lizot 2004: 183; Oliver et al. 1975: 146)
Tucano	kii	Cassava (Wilson and Dufour 2002)
Warao	aru (Romero-Figueroa 1997)	Flour (Heinen and Ruddle 1974[75]; Suárez 1968)
Awa-Cuaiquer	**pala**	Plantain (Carrera de la Torre 1991: 22)
Mai Brat	nait	Eat (Dol 2007: 3)
Piaroa	iɾe (Krute 1989)	Cassava (Juárez 2007)
Amanab	**fane** (Minch 1992: 111)	Food (Clark 1978: 291)
Choctaw	tãtʃiʔ	Maize (Haag and Willis 2001: 155)
Cherokee	kiʔa	Eat (John Rosh, p. c.)^c^

^a^The word for ‘tortilla’, /tɬaʃkali/, can be analyzed as /tɬa-iʃka-l-li/ obj.gen-cook-nact-abs.sg. (John J. Sullivan, p. c.). ^b^Although this source is not specifically about the Western Highland variety of Purepecha, Chamoreau considers Purepecha to be a single language (a language isolate). ^c^John Rosh (Cherokee Nation) confirmed to me that there is no single dominant type of staple food in the Cherokee tradition and different staples are consumed in different seasons.

The citations in the Meaning column refer to sources indicating each people’s single dominant staple food (or absence thereof). The people cited in the Meaning column may not be exactly the same people as the speakers of the language in the Language column. For example, Beck (2016), cited in the Meaning column at the row of Highland Totonac, indicates that the word for ‘tortilla’ can mean ‘food’ in Upper Necaxa Totonac. I have not been able to find information for Highland Totonac, but considering the geographical proximity of these two languages, it is probable that the main staple food is tortilla for Highland Totonac speakers as well. Thus I cited Beck (2016) to indicate that the staple food of Highland Totonac speakers is the tortilla. The cells of the Meaning column without citations are based on common knowledge.

All the morphemes are retrieved from the sources used in Joo Joo (2020), unless cited otherwise in the morpheme column. Morphemes that begin with /PA-/ are printed in bold. I could not find the corresponding morpheme for two languages, marked by a question mark. Moreover, the Eastern Huasteca Nahuatl word for ‘tortilla’ is polymorphemic, so it was left out. There are thus 63 morphemes in total.

Since both [+labial] phonemes and [+low] phonemes are very common, it is necessary to make a comparison to another list of morphemes. Among the 7,727 morphemes used in Joo’s (2020) study representing 66 languages and 100 meanings of the Leipzig-Jakarta List, I used the PanPhon database (Mortensen et al. 2016, last modified on 23 July 2020) to detect whether the first phoneme of the morpheme is [+labial] and/or the second phoneme is [+low].1The IPA transcription of (2020) and that of PanPhon are not identical, however. For example, the voiced bilabial prenasalized stop is transcribed as <mb> in Joo (2020), but as <b^n^> in PanPhon. Overall, approximately 1.4% of the phoneme tokens in the database of Joo (2020) are transcribed differently from the PanPhon database. In PanPhon, only low vowels (*a*-like vowels) have the [+low] feature, and not mid-low vowels such as /ʌ/. High rounded vowels, such as /u/ and /y/, have the [+labial] feature in PanPhon, but other rounded vowels, such as /o/ and /ø/, do not. I then compared the frequency of morphemes with the first [+labial] and/or the second [+low] phoneme in the two lists of phonemes.

## 4 Results and discussion

Table 2 compares the percentages (rounded to the first decimal) of morphemes whose first phoneme is [+labial] and/or whose second phoneme is [+low] of the two morpheme lists. We see that in all three cases, the percentage of the staple food morphemes is higher, especially when the two criteria (first [+labial] and second [+low]) are combined.

**Table 2:** Percentage of morphemes whose first phoneme is [+labial] and/or whose second phoneme is [+low].

	Staple food	Joo (2020)
First phoneme [+labial]	36.5%	22.8%
Second phoneme [+low]	46.0%	26.3%
Both	25.4%	6.7%

One explanation explaining the high frequency of /PA-/ in staple food morphemes is the standardization of baby talk words. Crosslinguistically, the word for ‘food’ or ‘to eat’ in baby talk (=parentese, motherese, infant-directed speech) is very often a reduplicated or semi-reduplicated syllable consisting of a bilabial consonant followed by a low vowel (Ferguson 1964; Joo 2021; Oswalt 1976; Weise 1903). And some of those baby talk terms may eventually be standardized into the default word for (staple) food, such as Saaroa /papaʔa/ ‘meat’ (Tsuchida 2009) or Korean /bab/ 밥 ‘cooked grain’ grain’ (Joo 2021).

In Table 3, I have listed the baby talk terms meaning ‘food/to eat’ and, as a comparison, those meaning ‘feces/to defecate’ in first ten of Table 1’s 66 sample languages. I limited to the first ten because baby talk words are often hard to retrieve from published sources. I see that all ‘food’ words begin with /PA-/, whereas none of the ‘feces’ words except for Mandarin /papa/ does (Also interesting to note is that all the ‘feces’ words except for Mandarin /papa/ bear at least one [+back] phoneme, such as /k/, /ħ/, or /ɯ/).

**Table 3:** Baby talk words for eating and defecating.

Language	Food/to eat	Feces/to defecate
Spanish	papa	kaka
Mandarin Chinese	–	papa
Yoruba	–	–
Egyptian spoken Arabic	mamm	daħħ
Javanese	maəm	ek-ek
Telugu	–	–
Turkish	mama	kaka
Japanese	mama	unko
Vietnamese	măm	–
Thai	mam-mam	ɯ

Joo (2021: 105) also shows that all the baby talk words for ‘food/to eat’ in 21 languages contain a bilabial consonant and/or a (near-)low vowel. Joo writes: “The association between /PA-/ and the concept of food or eating is quite straightforwardly iconic, since opening one’s mouth is the beginning and the most visible part of the eating process. Nurturers often perform the mouth-opening gesture to persuade the infants to eat their food, and that gesture may easily develop into baby-talk words, which in turn may be gradually ‘standardized’ and become part of the adult talk for ‘(the most common or important type of) food’” (pp. 104–105).

Another iconic motivation for the frequency of /PA-/ may be the association between labial sounds and softness. Staple foods, and foods in general, tend to be soft. Previous experiments (Kumagai 2020; Sakamoto and Watanabe 2018) demonstrated the perceptual association between bilabial consonants and softness, which the authors argue to be due to the soft texture of human lips. Joo’s (2020) typological study showed that [+labial] sounds are significantly frequent in morphemes representing ‘ash’ and ‘breast’, crosslinguistically. Ashes and breasts are, of course, soft in texture. This phonosemantic association, however, does not explain why [+low] phonemes are also frequent. Thus, it can only partially explain the frequency of /PA-/ in staple food morphemes.

Thus, this phonological similarity does not reflect a genealogical relatedness but rather an iconic motivation. And some of these words may have survived into adult talk, which helps to explain why /PA-/ is so common in adult talk morphemes representing staple food.

However, the standardization of baby talk cannot be the only mechanism responsible for the high tendency of /PA-/ in staple food terms. Some of the staple food morphemes beginning with /PA-/ shown in Table 1 are unlikely to be standardized baby talk words because they do not bear the reduplicated or semi-reduplicated form of most baby talk words, neither in their current form or at their earliest reconstructed stage, such as Spanish /pan/ ‘bread’ (<Latin *pānis* ‘(loaf of) bread’ < Proto-Italic *pa¯˘*st-ni* ‘loaf, cake’ < Proto-Indo-European ‘*peh*_2_*-s-* ‘to graze’, Vaan 2008: 443).

Another theory that may explain the high frequency of /PA-/ in staple food morphemes is the theory of the rebuilding of iconicity as set forth by Johansson and Carling (2015). According to this theory, a phonosemantic association can emerge not only via lexical creation but also via lexical change.

It is well-known that in spoken language deixis, proximal pronouns tend to bear high front vowels, whereas distal pronouns prefer low and/or back vowels (Johansson and Zlatev 2013; Tanz 1971; Woodworth 1991), such as English *th**i**s* and *th**a**t* or Indonesian *in**i*** ‘this’ and *it**u*** ‘that’. Johansson and Carling show that this phonosemantic association is not uniquely due to how demonstrative pronouns are created initially but also due to how they are changed subsequently.

For example, the Portuguese neuter proximal pronoun /iʃtu/ and neuter distal pronoun /akilu/ show a typical vowel distinction between the initial vowels /i/ and /a/. But this does not mean that /iʃtu/ and /akilu/ were created *ex nihilo* to express iconicity: /akilu/ is in fact a compound of the Latin expletive adverb *accu and demonstrative *ille* (Azevedo 2005: 159). Thus, the /a/ of /akilu/ does not represent a mimetic origin but rather a language change motivated by iconicity. Johansson and Carling demonstrated that a significant amount of Indo-European demonstrative pronouns have evolved in similar ways to ensure the iconic mapping between form and meaning.

In the light of this theory of the rebuilding of iconicity, the high frequency of /PA-/ in staple food morphemes does not necessarily mean that the morphemes that begin with /PA-/ and refer to a staple food must have done so from the beginning, rather it suggests the possibility that some morphemes gained their phonosemantic mapping between /PA-/ and the meaning of staple food after their creation via the rebuilding of iconicity.

As an example, one may argue that Spanish /pan/ ‘bread’ beginning with /PA-/ is only coincidental, since it originates from Proto-Indo-European *peh*_2_*-s-* ‘to graze’. But de Vaan (2008: 443) points out the difficulty of explaining the semantic shift from ‘to graze’ to ‘loaf, cake’ (in Proto-Italic *pa*¯˘st-ni*). Could this odd semantic change have been motivated by the rebuilding of iconicity?

Benczes (2020) hypothesized that sound symbolism may pressure semantic change, such as the semantic shift from Old English *bugan* ‘to bend’ to contemporary English *buxom*. Benczes suggests that this may be due to the presence of initial /b/ in related words like *breast* and *bosom*. As mentioned, [+labial] phonemes are frequent in words for ‘breast’ crosslinguistically (Joo 2020).

Similar to the case of *buxom* and Indo-European demonstrative pronouns, it is possible Spanish /pan/ has acquired its meaning of ‘bread’ from ‘to graze’ due to the rebuilding of the iconicity. Although most of the staple food morphemes in Table 1 do not have much historical information available, it is possible that some of the morphemes beginning with /PA-/ also acquired their meaning of staple food through the rebuilding of iconicity.

## 5 Conclusion

In this article, I have observed that many of the staple food morphemes begin with /PA-/, which I argue to be motivated by the iconic resemblance between the articulation of /PA-/ and the action of eating. I proposed two possible diachronic mechanisms behind this crosslinguistic tendency: (1) the standardization of baby talk into adult talk and (2) the rebuilding of iconicity. Since we use our mouths not only to speak but also to eat, it is no surprise that in some cases, our way of speaking may resemble our way of eating.

## Data availability statement

The data generated and analyzed during this study are available in this article as Table 1.
